# Mining the microbiome of Lake Afdera to gain insights into microbial diversity and biosynthetic potential

**DOI:** 10.1093/femsmc/xtae008

**Published:** 2024-03-07

**Authors:** Ermias Sissay Balcha, Michael C Macey, Mesfin Tafesse Gemeda, Barbara Cavalazzi, Adugna Abdi Woldesemayat

**Affiliations:** School of Medical Laboratory Science, College of Health Sciences, Hawassa University, 16417, Hawassa, Ethiopia; Biotechnology and Bioprocess Center of Excellence, College of Biological and Chemical Engineering, Addis Ababa Science and Technology University, 16417, Addis Ababa, Ethiopia; Astrobiology OU, School of Environment, Earth and Ecosystem Sciences, The Open University, Milton Keynes, MK7 6AA, United Kingdom; Biotechnology and Bioprocess Center of Excellence, College of Biological and Chemical Engineering, Addis Ababa Science and Technology University, 16417, Addis Ababa, Ethiopia; Dipartimento di Scienze Biologiche, Geologiche e Ambientali, Università di Bologna, Bologna, Italy; Department of Geology, University of Johannesburg, Johannesburg, South Africa; Biotechnology and Bioprocess Center of Excellence, College of Biological and Chemical Engineering, Addis Ababa Science and Technology University, 16417, Addis Ababa, Ethiopia

**Keywords:** biosynthetic gene clusters, Lake Afdera, secondary metabolites, metagenomics, shotgun sequencing, microbiome

## Abstract

Microorganisms inhabiting hypersaline environments have received significant attention due to their ability to thrive under poly-extreme conditions, including high salinity, elevated temperatures and heavy metal stress. They are believed to possess biosynthetic gene clusters (BGCs) that encode secondary metabolites as survival strategy and offer potential biotechnological applications. In this study, we mined BGCs in shotgun metagenomic sequences generated from Lake Afdera, a hypersaline lake in the Afar Depression, Ethiopia. The microbiome of Lake Afdera is predominantly bacterial, with *Acinetobacter* (18.6%) and *Pseudomonas* (11.8%) being ubiquitously detected. A total of 94 distinct BGCs were identified in the metagenomic data. These BGCs are found to encode secondary metabolites with two main categories of functions: (i) potential pharmaceutical applications (nonribosomal peptide synthase NRPs, polyketide synthase, others) and (ii) miscellaneous roles conferring adaptation to extreme environment (bacteriocins, ectoine, others). Notably, NRPs (20.6%) and bacteriocins (10.6%) were the most abundant. Furthermore, our metagenomic analysis predicted gene clusters that enable microbes to defend against a wide range of toxic metals, oxidative stress and osmotic stress. These findings suggest that Lake Afdera is a rich biological reservoir, with the predicted BGCs playing critical role in the survival and adaptation of extremophiles.

## Introduction

The microbiome of hypersaline environments is consists of specialized microorganisms known as halophiles. (Waditee-Sirisattha et al. 2016). Halophiles occupy a diverse ecological niches, including microbial mats, saline soils, hypersaline soda lakes, brine pools, and salt Lakes (Weimer and Rompato 2009, Chen et al. 2020). To survive in these environments, halophiles have developed adaptations to multiple stressors, such as ionic stress, osmotic stress, desiccation stress and carbon-poor conditions (Corral et al. 2020). These adaptations include various genetic mechanisms, for example, efflux pumps, which help microbes to acclimatize to their environment (Oren 2015). Additionally, many survival mechanisms involve the production of secondary metabolites, which potentially serve as sources of biotechnologically valuable molecules with a wide range of applications. For instance, halophiles produce stable enzymes beneficial in different industrial processes, ranging from biopolymer production to bioremediation (Makarova et al. 2019), creation of macromolecule stabilizers and biofertilizers (Dassarma et al. 2010, Amoozegar et al. 2017) to a large number of applications in fermented food products. (Yin et al. 2015, Kiadehi et al. 2018). Despite their commercial potential, halophiles remain relatively underexplored in terms of their capacity to produce antimicrobial and anticancer drugs (Charlesworth and Burns 2015, Corral et al. 2020).

Biosynthetic gene clusters (BGCs) are a locally clustered group of two or more genes that confers a competitive advantages to microorganisms by encoding secondary metabolites (Medema et al. 2015). These clusters encompass a variety of chemical and biological groups, including non-ribosomal peptide synthetases (NRPS), polyketide synthases (PKS), terpenes, and bacteriocins (Wang et al. 2019). NRPS and PKS are particularly important targets for natural product discovery as they are known to synthesize a diverse array of antimicrobials and pharmaceutical products (Tillett et al. 2000, Wang et al. 2014, Chen et al. 2020). The condensation (C) and ketosynthase (KS) domains within these clusters serve as conserved genomic markers, essential to distinguish between different NRPS/PKS natural product pathways (Ziemert et al. 2012). Previously, the detection of these pathways has led to the identification of compounds with unique chemistry, such as lactocillin (Donia et al. 2014) and salinilactam (Udwary et al. 2007) antibiotics. Currently, research on undiscovered BGCs involves an increasing focus on genomic and metagenomic analysis (Makarova et al. 2019). These approaches enhance the reconstruction of near-complete genomes *de novo*, enabling the potential recovery of novel BGCs in both culturable and uncultivable microorganisms. One such interesting niche to discover bioactive compounds using this method is the brine pool of Afdera. The microbiology of this lake remains unexplored although halophilic microorganisms have been detected by molecular studies carried out in commercial salt from the salterns bordering the lake (Gibtan et al. 2017).

**Figure 1.**
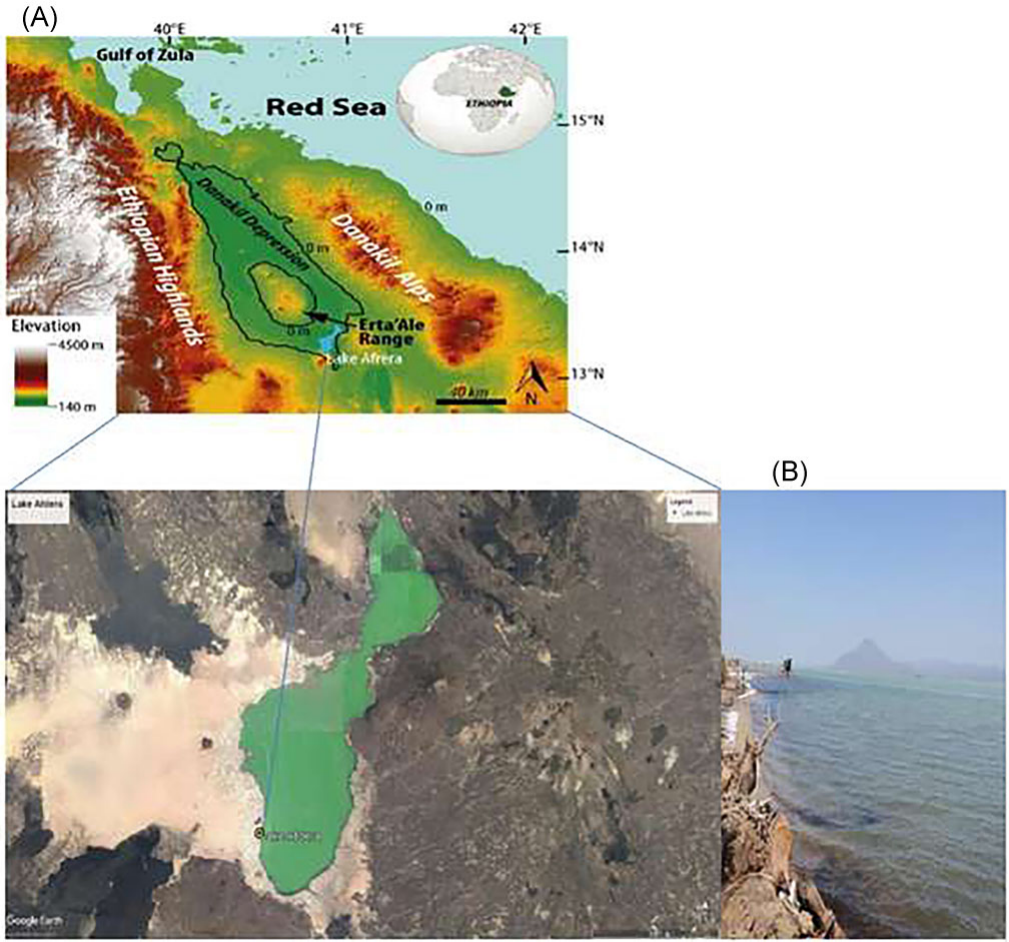
(A) Satellite image of Lake Afdera, Afar Depression, northern Ethiopia and (B) Panoramic view showing the sampling location site

The brines in the Afar depression, including those in Lake Afdera, are enriched with different metals, such as lithium, which is of interest for industrial applications and represents an additional selective pressure (Bekele and Schmerold 2020). Lake Afdera, the largest of brine lakes in the Afar depression, is characterized for its maximum depth (80 m), volume (2.4 km^3^) and elevation (-112 m) (Fig. 1). Previous studies have isolated and characterized thermostable amylase-producing bacteria (Yassin et al. 2021) and fungi (Welday et al. 2014) from soil samples of Afdera. However, no studies have yet explored the potential significance of BGCs in this lake. The present study aims to determine the diversity of BGCs from the microbial populations recovered from Lake Afdera to provide insights into their genetic potential and identify the adaptation functions of these BGCs in this ecosystem. To achieve this, we conducted in-depth analyses of metagenome assembled genomes (MAGs) using antibiotics and Secondary Metabolites Analysis SHell (antiSMASH), BActeriocin GEnome mining tooL (BAGEL4) and Natural Product Domain Seeker (NaPDoS) pipelines. We present the microbiome composition of Lake Afdera. Additionally, we have identified biotechnologically significant BGCs in the metagenomic data that are proposed to play important roles in adaptation to poly-extreme environments.

## Materials and methods

### Sampling and measurement of physicochemical parameters

During two separate field trips, triplicate water (4000 mL) samples were collected directly from the edge of Lake Afdera. The first field trip took place in April 2021 at 0703016 E/1462263 N and samples were collected across transects of the lake. In the second field trip in October 2021, additional samples were collected at 0703009 E/1462256 N. Physicochemical parameters (pH, total dissolved solid (TDS), temperature and salinity) were measured *in situ* using a portable refractometer (HI-9829–02 advanced portable multi-parameter pH/ISE/EC/DO/Turbidity waterproof meter, Eden Way, United Kingdom). All sampling points and mapping data were georeferenced using a Garmin® handheld GPSMAP64. The samples were collected under stringent aseptic conditions and sterilized glass bottles, which were then capped and sealed with parafilm tape. Bottles were transported in an ice box and stored at −20°C until further analysis. Samples for culture assays were kept at 4°C.

### Determination of elemental composition

The elemental composition of the lake was determined via use of an Inductively Coupled Plasma Optical Emission Spectrometer (ICP-OES), (Agilent 5100 SVDV ICP- OES from the USA, conforming to ES ISO 11885:2007 standards). The detection limit was 0.01 μg L^−1^. The standard working parameters were selected and a published outlined procedures were followed (Wiel 2003). Prior to analysis, the samples (50 mL) were subjected to digestion at 80°C with 10 mL of nitric acid, cooled, filtered and diluted to 100 mL with distill water.

### Enrichment media and isolation of halophilic bacteria

Culture media was formulated based on a classical halophile mineral growth medium, incorporating the physico-chemical characteristics of Lake Afdera's environment. This media design followed a modified published method (Kiki 2016, Belilla et al. 2019). A nutrient broth (NB 1090 media), using a ratio of 10:90 samples to broth, was employed. The nutrient broth was prepared using artificial sea water, comprising of NaCl 100 g, MgCl_2_ 8 g, MgSO_4_ 20 g, CaCl_2_ 0.5 g, KCl 2.5 g, FeSO_4_ 1 g, dissolved in sterilized distilled water to achieve a final volume of 1000 ml. The mixture was then autoclaved, cooled and poured into sterilized falcon tubes containing the samples, the falcon tube containing the mix were subjected to incubation at 37°C for 7 days in an orbital shaker. For bacterial isolation, 100 µL of the broth was streaked onto an agar medium composed of starch 15 g, glucose 2.5 g, yeast extract 2.5 g, agar 20 g and sterilized artificial seawater 1000 ml, pH adjusted at 5.8. The plates were incubated at 37ºC for a period of 7–14 days.

### Genomic DNA extraction and 16S rRNA gene sequencing

Genomic DNA was extracted using GeneMark bacterial DNA purification kit. Appropriate liquid media cultures for bacterial DNA extractions were prepared and incubated at 37°C, 150 rpm for 7 days. The extraction was completed following the manufacturer's instructions provided in the kit. The 16S rRNA gene was sequenced using ABI 3730XL platform, service provided by Inqaba Biotec, Pretoria, South Africa. Sequence chromatogram analysis was performed using FinchTV analysis software. Taxonomic identification was conducted using the NCBI BLASTN server. A phylogenetic tree was constructed using maximum likelihood method using Kimura-2 parameter model.

### Environmental DNA extraction and metagenomic sequencing

The water samples (1000 mL) were sequentially filtered through 0.45 and 0.22 µm GE® polycarbonate filter membrane. The membranes from 0.22 µm were cut into small pieces in sterilized condition and DNA extraction was performed using an optimized CTAB method (Zhou et al. 1996). The DNA extractions were performed in triplicates and replicates were later pooled prior to metagenome sequencing. DNA quality and quantity was examined with a Thermo Scientific NanoDrop 3300 Fluorospectrometer (Thermofisher Scientific, USA). The extracted DNA was randomly sheared into short fragments and ligated with Illumina adapters to construct a library. The libraries were pooled, barcoded and subsequently shotgun sequenced on one lane of a flow cell using a 150 bp paired-end run on a NovaSeq PE150 instrument (Illumina) at Novo-gene (Hong Kong). The sequences were de-multiplexed using Cassava v.2.0 and FastQC was used for quality control checks on the sequence composition of paired-end raw reads. Trimmomatic v0.36 (Q-value ≤ 38; N >10 bp; reads overlap with adapter >15 bp) was employed to remove low quality bases and any adapter contamination.

### Assembly, metagenome assembly of genomes (binning) and annotation

The assembly of the reads was initially conducted using MEGAHIT (Li et al. 2015). Scaffolds containing “N” were removed and scaftigs were subsequently formed (Mende et al. 2012, Nielsen et al. 2014). The cleaned reads were then mapped to assembled scaftigs using Bowtie2 and the unutilized paired end reads were collected (Langmead and Salzberg 2012). Mixed assembly was carried out on unutilized reads and after which reads shorter than 500 bp were trimmed from the scaftigs and the mixed assembled units (Li et al. 2014). The assembled contigs were further binned into metagenome assembled genomes (MAGs) using the method describe by Yang and co-authors (Yang et al. 2021). MAGs (binning) was conducted using MetaBAT2 (Kang et al. 2019), CONCOCT (Alneberg et al. 2014), and MaxBin (Wu et al. 2016). The retrieved MAGs were pooled with DAS Tool (v1.1.1) (Sieber et al. 2018) and their completeness and contamination were assessed using CheckM (≥ 80% completeness and ≤ 10% contamination) (Parks et al. 2015). The metagenome was annotated using NCBI GenBank annotation pipeline (Altschup et al. 1990) and RAST (Rapid Annotation using Subsystem Technology) tools, employing the classic RAST annotation scheme (Aziz et al. 2008). Furthermore, functional classification of the predicted genes was conducted using the Cluster of Orthologous Groups (COG) framework (Tatusov et al. 2003).

### Taxonomic Assignment of Contigs

The taxonomic diversity of the assembled contigs was performed by comparing metagenomic reads based on sequence or phylogenetic similarity to the database sequence of taxonomically informative gene families (microNR database) (Buchfink et al. 2014). The taxonomic annotation of each metagenomic homolog was then carried out using MEtaGenome Analyzer community edition (MEGAN) (Huson et al. 2011). MEGAN allocated the reads onto the NCBI taxonomy using settings of the Lowest Common Ancestor (LCA) algorithm. Tree file extracted from MEGAN was uploaded to an online Interactive Tree Of Life (iTOL) version 5.0 (Letunic and Bork 2021) and circular phylogenetic tree was constructed.

### Detection of BGCs

The MAGs were subjected to antiSMASH 6.0.1 (Blin et al. 2021) to mine BGCs and contigs of size equal to or larger than 1000 bp were utilized. The RAST-web tool server and NCBI Gen- Bank annotation pipeline were utilized to identify various proteins and genes responsible for adaptation to extreme environment. The identified BGCs were compiled, and a stacked bar chart was generated using R studio, with visualization created in ggplot2 and R Color Brewer packages.

### Detection of bacteriocins and domains of NRPS and KS

BAGEL4 was used to assess the bacteriocin and ribosomally synthesized and posttranslational modified peptides (RiPPs) within the investigated MAGs (Heel et al. 2018). Potential clusters and annotated classifications were identified as areas of interest (AOI). In addition, NaPdoS was used to search potential known natural product biosynthetic domains, specifically C and KS, by comparing them to a domain database of previously characterized natural products (Ziemert et al. 2012). Subsequently, a circular phylogenetic tree was constructed for all C and KS domains using NaPDoS. The resulting trees were visually presented and annotated using the online interactive Tree of Life (iToL) version 5.0 (Letunic and Bork 2021).

## Results

### Physico-chemical measurements, elemental composition and enrichment media

Physico-chemical measurements (Table 1) indicate that Lake Afdera is predominantly saline, with a slightly acidic pH. The GPS meter show that the lake is one of the lowest land areas in Africa. The elemental composition revealed that the lake is contaminated with heavy metals (Table 1), including iron (Fe), lead (Pb), zinc (Zn), and nickel (Ni). Additionally, appreciable amount (15.7 mg/L) of lithium (Li) was detected in the lake.

**Table 1.** Physico-chemical measurements and elemental content (mg/L) of Lake Afdera, water samples taken in April, 2021.

Measured parameters	Measured value
Temperature (°C)	36
pH	5.89
Salinity (g/L)	137
Altitude (m)	-112
Na	38 500
Ca	13 900
Mg	1090
Cl	1590
Mn	0.618
Cu	0.841
Pb	0.018
Zn	0.013
Ni	1.350
Fe	2.160
Cd	0.001
Li	15.7
Sr	183

**Figure 2.**
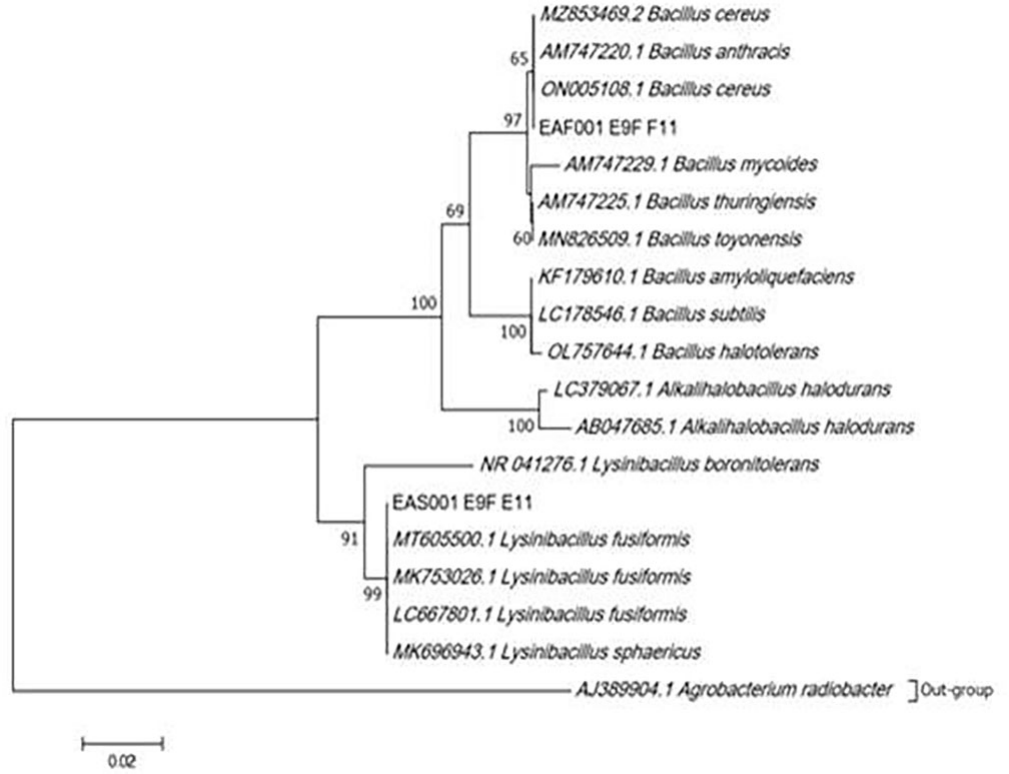
Phylogenetic tree constructed using maximum likelihood method using Kimura-2 parameter model. The robustness of tree was evaluated by Bootstrap method (1000 replication). Only bootstrap values greater than 50% are shown, and the scale bar indicates 0.02 substitutions per site.

In this study, bacterial species similar to *Bacillus* were isolated using an optimized halophilic enrichment media called NB 1090. The sequencing results from 16S rRNA gene identified EAS001 as closely related to *Lysinibaccilus fusilformis* and EAF001 as *Bacillus cereus*. The phylogenetic tree also displayed closely related species (Fig. 2).

### Metagenome data analysis and microbial community compositions

A total of 45081590 reads were generated with GC content of 56% (Table 2). These reads were subsequently assembled into 93220 contig sequences, with a total contig length of 108548798 bp (Table 2). From the metagenomic assembly (binning), 17 MAGs were generated and following inspection using CheckM, ten candidate MAGs representing different assigned phyla were selected. These were chosen for detection and investigation of BGCs and environmental adaptation mechanisms (Table S7).

**Table 2.** Metagenome sequence analysis and assembly data.

Sequencing parameters	Quantified Value
Library insert size (bp)	350
DNA concentration used for sequencing (ng/µl)	7.8
Length of single read (bp)	150
Total number of raw reads	45081590
Total number of cleaned reads (Mbp)	6759.12
GC content (%)	56
No. of contigs	93220
Longest contig length (bp)	176724
N50 (bp)	1212
Total length (bp)	108548798

**Figure 3.**
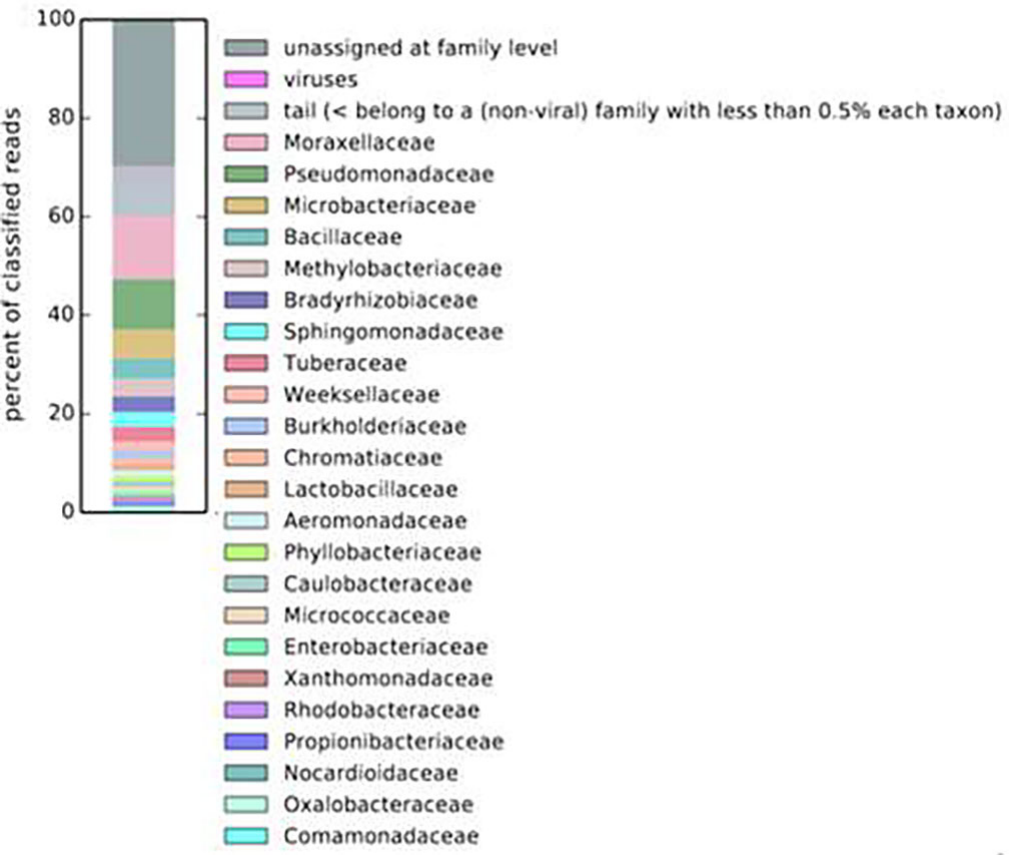
Stacked bar plot showing the relative abundance of microbial communities in brine pool habitats of Lake Afdera, according to family-level taxonomic distribution.

**Figure 4.**
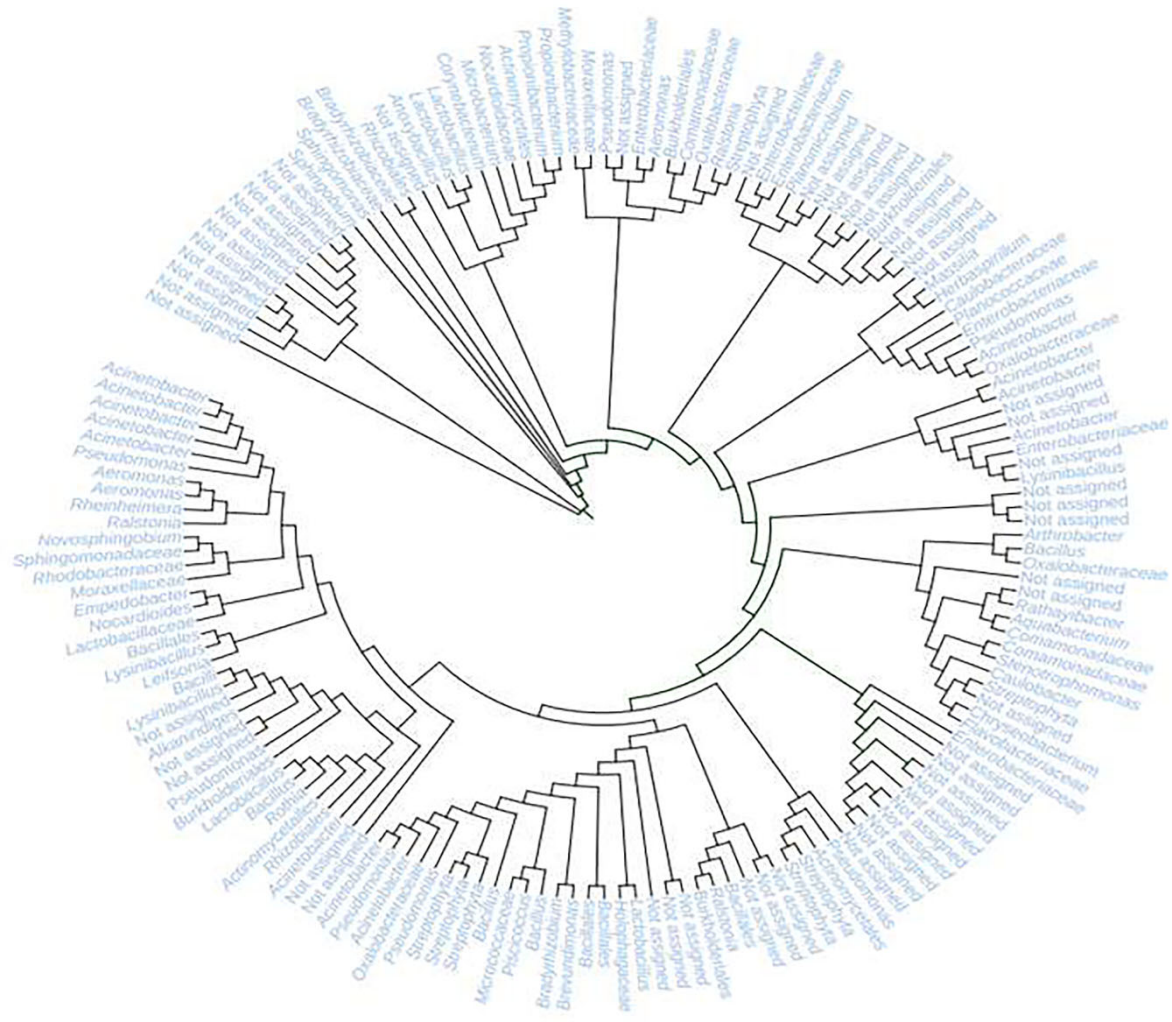
Circular phylogeny tree for metagenomically detected microbes from water samples collected from Lake Afdera

The microbial composition of the metagenome was dominated by bacteria (94.932%), followed by eukarya (0.3663%), archaea (0.1913%) and 4.5103% of the reads were unclassified taxa (Fig. 3). The phyla detected in the sampling sites included Pseudomonadota (63.6%), Actinomycetota (13.9%), Bacilliota (7.8%), Bacteroidota (2.6%) and Cyanobacteria (2%). Gammaproteobacteria (42.9%) and Alphaproteobacteria (16.8%) were the most identified classes within the phylum Pseudomonadota, followed by Actinobacteria (13.9%), Bacilli (7.7%) Betaproteobacteria (3.1%) and Flavobacteriia (2.4%). The genus with the highest occurrence across the sampling sites was *Acinetobacter* (18.6%), followed by *Pseudomonas* (11.8%), which represents the second most frequent lineage within the Gammaproteobacteria. The remaining genera identified in the microbiome of Lake Afdera included *Microbacterium* (7.6%), *Bacillus* (4.7%), and *Methylobacterium* (4.1%) (Fig. 4). Among other recognized bacterial lineages, members of Candidatus Kaiserbacteria, Mucoromycota, Basidiomycota, Ascomycota, Acidobacteria were found in minor amounts (<1% of reads).

Additionally, the archaeal population was represented in the metagenome sequences, with archaeal phyla such as Candidatus Nanohaloarchaeota, Crenarchaeota, and Euryarchaeota being detected. In addition to these phyla, we identified a small percentage (less than 1%) of the community at the class level. These include Nanohaloarchaea, Thermoprotei, Halobacteria, Methanobacteria, Methanomicrobia, and Thermococci.

### Secondary metabolism BGCs

A total of 68 BGCs were detected from 10 MAGs using antiSMASH (Table 3 and Table S1). Among these, 20.6% of the BGCs encoded for non-ribosomal peptide-synthetase (NRPS), showing 100% gene similarity to bacitracin and lichenysin from the MIBIG most similarity cluster database index (Table S1).

**Table 3.** The BGC types detected in the water samples of Lake Afdera and their respective biotechnological and industrial applications.

S.no.	Taxonomy	BGCs type	Biotechnological applications	References
MAG 1	Actinomycetota (phylum)	LAP	Narrow-spectrum antibacterial	(Kloosterman et al. 2020)
		β-lactone	Antimicrobial and anti-cancer drugs	(Robinson et al. 2019)
		Bacteriocins	Antibacterial activity	(Yang et al. 2014)
		CDPS	Antimicrobial, anticancer activity	(Singh et al. 2021)
MAG 2	Bacilliota (phylum)	Lanthipeptide	Antimicrobial	(Montalbán-López et al. 2021)
		Lassopeptide	Antimicrobial and anticancer	(Cheng and Hua 2020)
		Thiopeptide	Antibacterial activities	(Chan and Burrows 2021)
MAG 3	Cyanobacteria (phylum)	Terpene	Antimalarial, anti-cancer, antiviral, antimicrobial, anti-inflammatory, anti-diabetic activity	(Cox-Georgian et al. 2019)
MAG 4	Gammaproteobacteria (class)	Ranthipeptide	Resistance to stressors	(Russell et al. 2020)
MAG 5	Betaproteobacteria (class)	NAGGN	Industrial application, Osmoprotectants	(Sagot et al. 2010)
MAG 6	Alphaproteobacteria (class)	RiPP like	Antimicrobial	(Montalbán-López et al. 2021)
MAG 7	Bacteroidota (phylum)	Ectoine	Industrial application, Osmoprotectants	(Kuhlmann et al. 2011)
MAG 8	Flavobacteria (class)	Redox-cofactor	Maintain cellular redox balance	(Xie and Zhang 2022)
MAG 9	Gammaproteobacteria (class)	Arylpolyene	Antioxidative products	(Ziko et al. 2019)
MAG 10	Pseudomonadota (phylum)	NRPs	Antimicrobial, immunosuppressant and anticancer drugs	(Felnagle et al. 2008)

**Figure 5.**
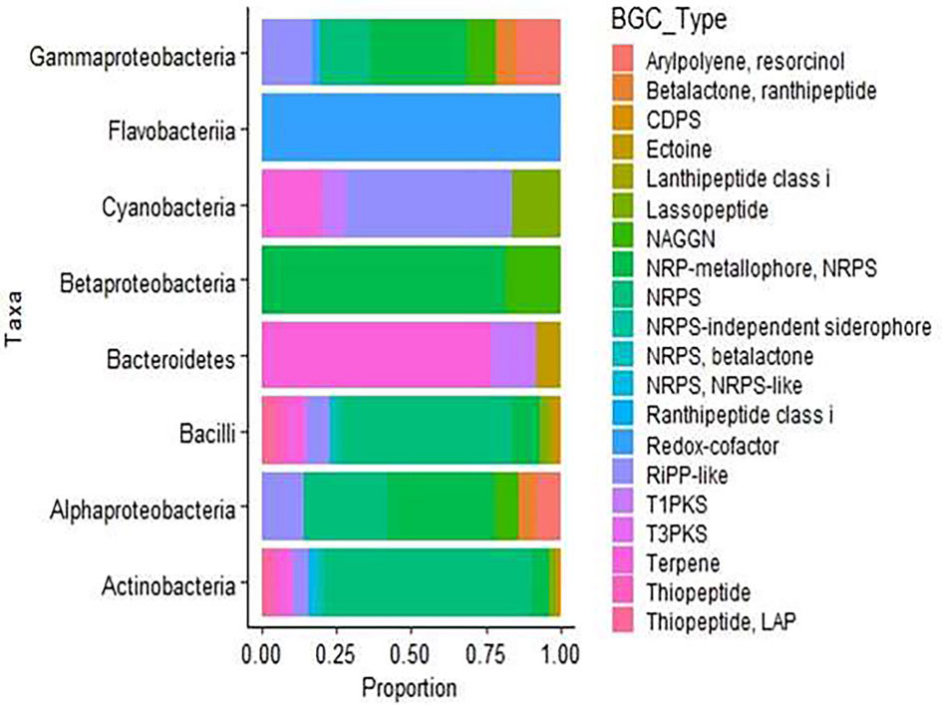
Stacked bar chart of various BGC types identified by antiSMASH in the MAGs of different assigned taxa at class level and only taxa with predicted BGCs was plotted. Gene clusters are arranged left to right based on average proportion contribution on each MAGs

Additionally, 13.2% BGCs encoded for NRP-metallophore, showing 100% genes similarity to bacillibactin from the same database index were identified. Other BGCs included 10.3% RiPP like and 5.9% each of thiopeptide-LAP hybrid cluster, terpene, N-Acetylglutaminylglutamine amide (NAGGN), arylpolyene, resorcinol clusters, Linear azol(ine)e‐containing peptides (LAP) was detected in the MAGs. Further, 1.5% ectoine and 2.9% cyclodipeptide synthase (CDPS) BGCs were identified in MAGs (Fig. 5). Among the MAGs, Actinomycetota exhibited the highest number of BGCs, closely followed by Bacilliota (Supplementary Table S1).

**Figure 6.**
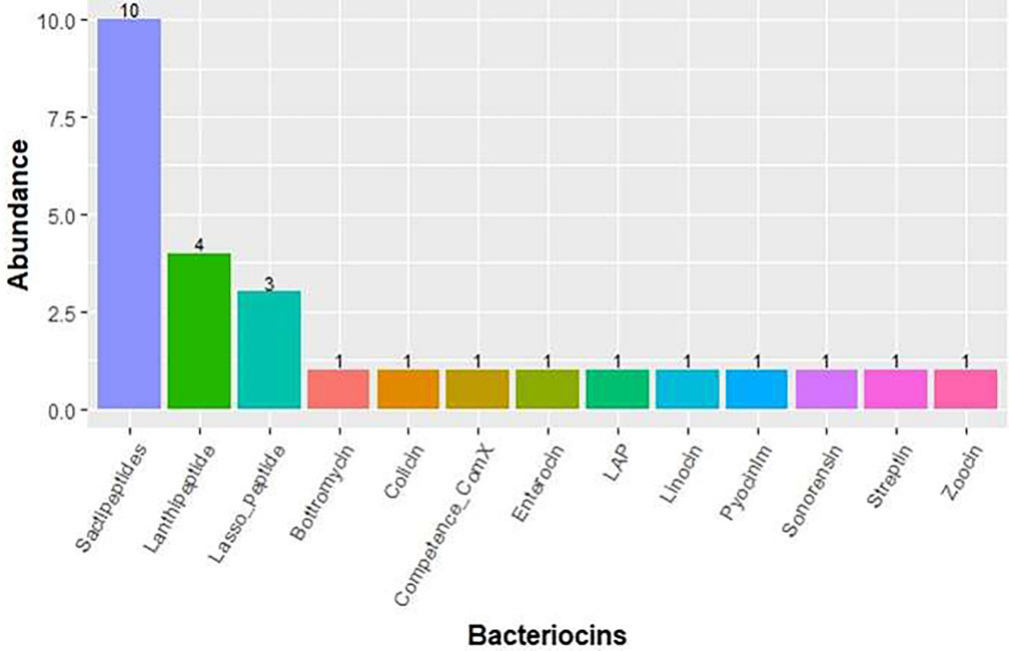
Bar plot shows abundance of bacteriocin cluster types and their subsequent subclasses predicted by BAGEL4 from the recovered MAGs. Sactipeptides was found to be the most abundant predicted bacteriocin class.

A total of 26 bacteriocins BGCs were also identified via BAGEL4 (Fig. 6). In this study, sactipeptides (40%) are the most prevalent bacteriocin clusters followed by Lanthipeptide (16%) and Lasso peptide (12%). MAGs assigned to Bacilliota (eight), Cyanobacteria (seven) and Bacteroidota (six) abundantly contained genes that encode a range of bacteriocins (Supplementary Table S2).

### Analysis of KS and C Domains

**Figure 7.**
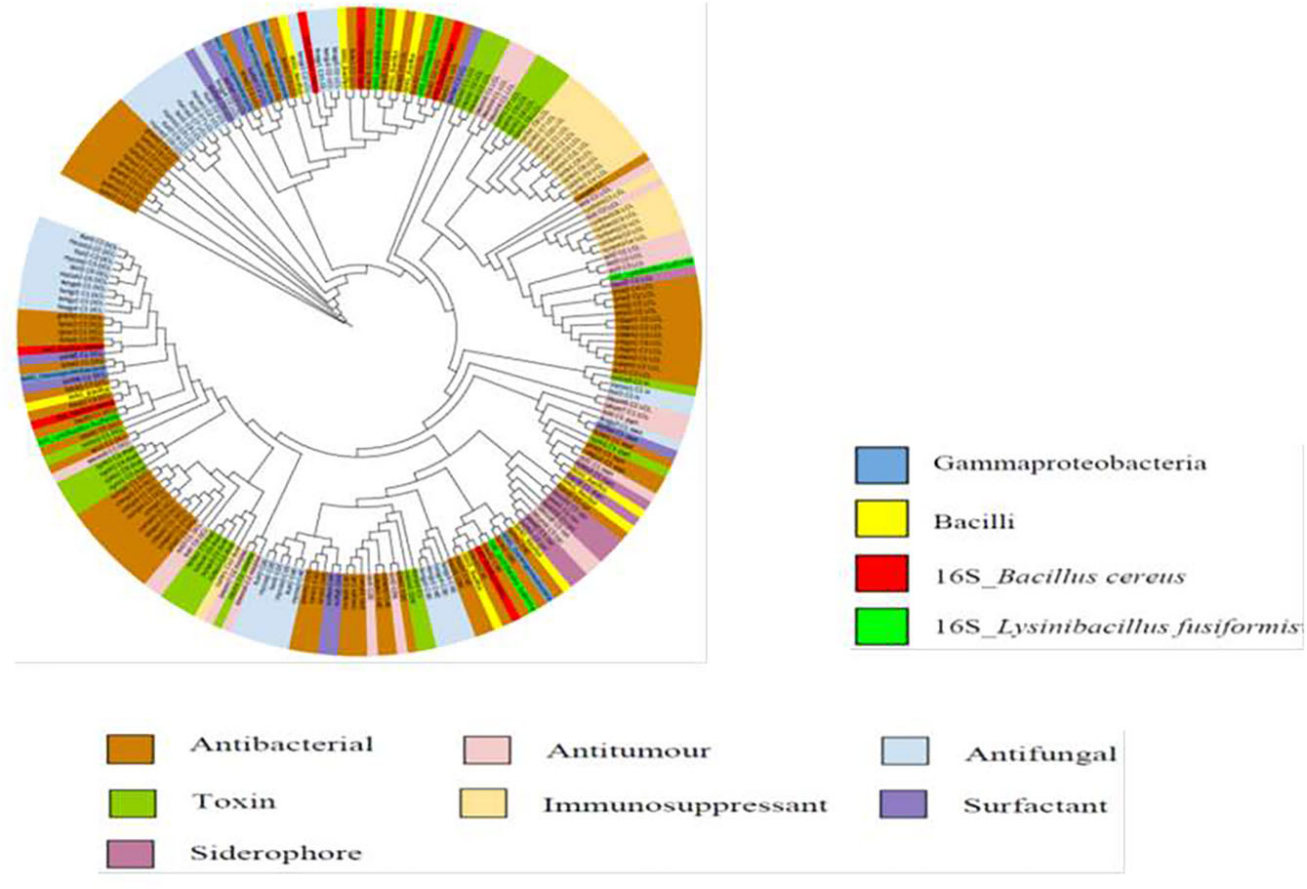
Phylogenetic tree constructed using maximum likelihood method to analyze condensation (C) domains against the NaPDoS domain database. The outer ring of the tree represents natural products, which are shaded according to their bioactivity. Various C domains aligned most closely to pathways encoding for antibacterial and antifungal compounds.

Compounds such as fengycin, bacitracin and lichenysin contributed over 80% of the total C domains detected. In addition, several other biologically active compounds, like syringomycin, complestatin, mycosubtilin, microcystin, nostopeptolide, bacillibactin, pyoverdine were detected in the MAGs assigned for Bacilli, Gammaproteobacteria and Cyanobacteria (Fig. 7, Table S4).

**Figure 8.**
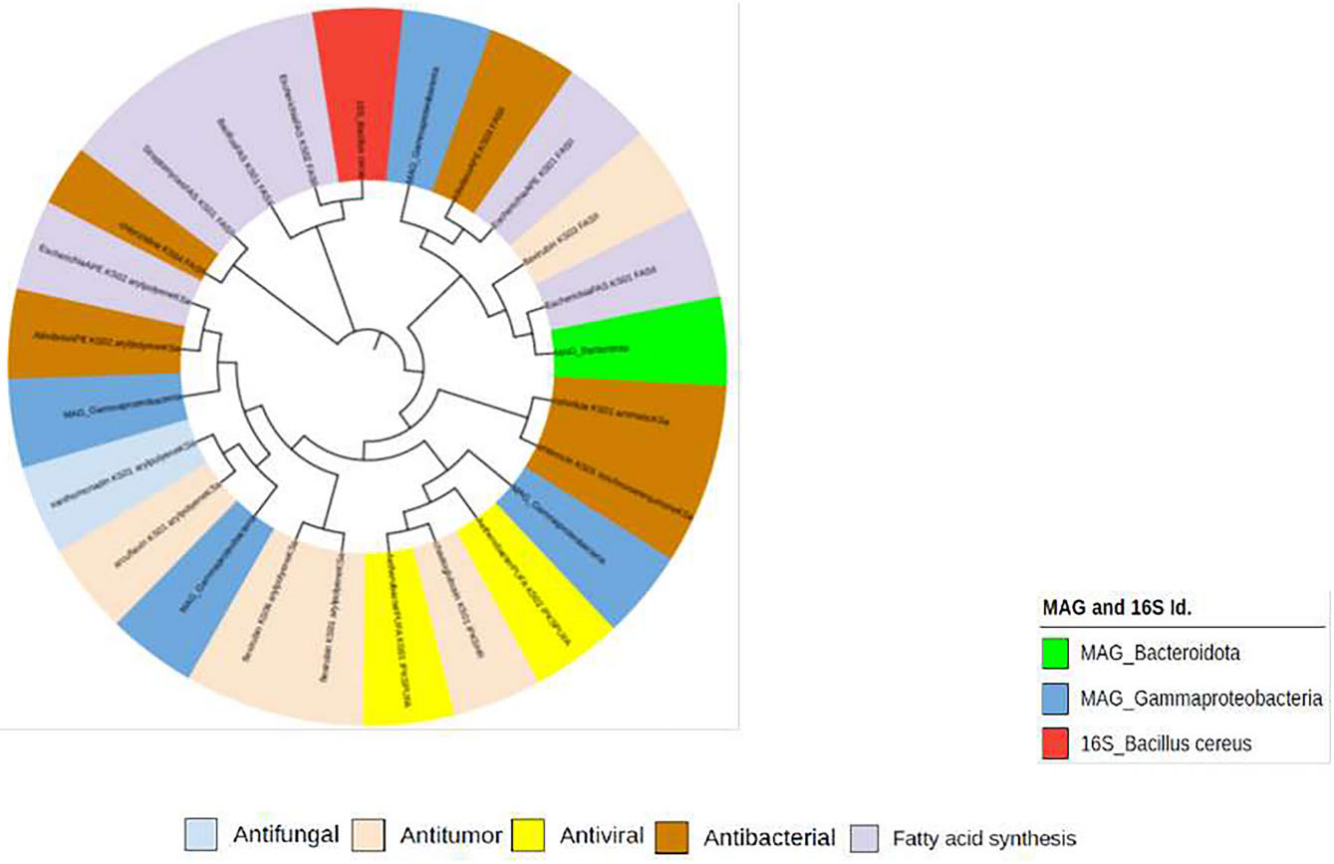
Phylogenetic tree constructed to analyze ketosynthase (KS) domains against the NaPDoS domain database using maximum likelihood method. The outer ring of the tree represents natural products, which are shaded according to their bioactivity and domains also color coded. Most KS domains are aligned closely to fatty acid synthesis, antitumor and antibacterial compounds. Novel Biosynthetic Gene Clusters offer key role to discover unique natural products in extreme Halophilic environments

The KS domain from the analyzed MAGs aligned closely with aryl polyene, chaetoglobosin, iterative cis-AT and modular cis-AT as well as Fatty acid synthesis (FAS) (Fig. 8, Supplementary Table S3 and S5).

### Genomic insights into microbial survivability mechanisms

Through annotation using NCBI GenBank pipeline, RAST tool and COG database, we identified various putative microbial genes, proteins and compounds associated with metal resistance and adaptation to the metal-rich environments of Lake Afdera. Genes encoding copper oxidases (*copA*) and copper resistance proteins (*copB, copC, copD, copG*) were detected (Table S6). Additionally, the presence of genes such as ferric uptake regulator (*fur*) and Fe-bacillibactin uptake systems (*FeuABC*) suggests regulatory mechanisms for managing excess concentration of iron (Tables S6, S8). Our sequence analysis also revealed multiple gene clusters encoding proteins involved in mercury resistance, including mercuric ion reductase (*merA*) and genes regulating transcriptional mercuric reductase (Table S6). Furthermore, multi-heavy metal efflux proteins such as magnesium- cobalt efflux protein CorC and the cobalt-zinc-cadmium resistance protein CzcD were detected (Tables S6, S8).

Multiple gene clusters combating oxidative stress were also identified, including gor for glutathione reductase, *grxA* for glutaredoxin 3, *rbr* for rubredoxin and other antioxidant related genes (Table S6, S8). Moreover, genes encoding enzymes that neutralize oxidative stress, such as superoxide dismutase, glutathione peroxidase, glutathione synthetase, gamma-glutamyltranspeptidase, glutamate-cysteine ligase, glutathione S-transferase, Alkyl hydroperoxide reductase subunit C-like protein were identified (Table S6). We also found multiple copies of putative genes encoding proteins responsive to osmotic stress, including opuAC for choline, glycine/betaine uptake and biosynthesis; *soxA* for sarcosine oxidase; *betT* for High-affinity choline uptake system and various osmoprotectants (e.g. *proW*) (Tables S6, S8). Further, the genome analysis revealed predicted compounds such as ectoines and osmoprotectant peptides (N-acetylglutaminylglutamine amide, NAGGN) (Table S1).

## Discussion

The advent of genome mining tools for studying microbial genomes has led to the widespread identification of BGCs across the bacterial domain (Cimermancic et al. 2014). BGCs are distributed in various gene cluster families and play crucial role in providing microbial communities with the ability to adapt to extreme environments (Wang et al. 2019). Herein, we present the first metagenome mining study of BGCs from understudied microorganisms in Lake Afdera, shedding lights into their chemical potential as well as their adaptation mechanisms within the poly-extreme habitat. Moreover, we have developed an inexpensive classical halophile mineral growth medium, NB 1090, by modifying a published method (Kiki 2016, Belilla et al. 2019). Such isolation media may be instrumental for cultivation of microbes from hypersaline environments. Our optimized method enriched microbial species related to *B. cereus* and *L. fusiformis* as evidenced from the sequenced 16S rRNA gene data. This finding aligns with previous research on Afdera soil, which reported thermostable amylases from culturally isolated *Bacillus* species (Yassin et al. 2021).

The microbiome of Lake Afdera mainly consists of the bacterial phyla such as Pseudomonadota, Actinomycetota, Bacilliota, Bacteroidota and Cyanobacteria. This finding is consistent with similar studies conducted in hyper-arid regions of Atacama desert, where these phyla were frequently observed (Azua-Bustos et al. 2015, Orellana et al. 2018). Additionally, a 16S rRNA gene survey of commercial salts extracted from Lake Afdera revealed an abundance of Pseudomonadota, Bacteroidota, Actinomycetota and Bacilliota (Gibtan et al. 2017). A broad bacterial (and to a lesser extent, archaeal) diversity was identified in the saline rich multi-extreme environment of the Afar Depression. This diversity might result from multiple independent adaptation mechanisms within the archaeal community, which appear to contrast with the extensive molecular adaptations observed in the bacterial domain (Hallsworth et al. 2007, Stevenson et al. 2015, Lee et al. 2018). An example of such an adaptation is the intracellular accumulation of K^+^ (the ‘salt-in’ strategy), which must function in conjunction with intracellular proteins adapted to these harsh conditions. Prior to the present study, it has not been shown whether the identified phylum from Lake Afdera possess biosynthetic capacity for natural compound discovery.

The MAGs were thoroughly analyzed using antiSMASH 6.0.1 to predict BGCs and a total of 68 BGCs were detected with NRPS, bacteriocins and NRP-metallophore being the most abundant among them. Among the identified taxonomies, the highest number of BGCs was detected in the MAGs associated with Actinomycetota and Bacilliota, supporting their reported biosynthetic capacity (Fig. 5). An antiSMASH survey of these phyla harbored the most diverse BGCs, to mention few NRPS, PKS, saccharide, β-lactone, RiPP like, CDPS (Table S1). Saccharides enable bacteria to form biofilms thereby providing adaptation mechanisms from toxins and dehydration (Wolferen et al. 2018). CDPS and β‐lactones exhibited a broad range of biological activities, including antimicrobial and anticancer activities, making them promising candidates for developing new pharmaceuticals to combat the growing antibiotic resistance crisis (Li and Rebuffat 2020, Singh et al. 2021). Other identified BGCs and their associated functions further underscores the microbiome's potential in Lake Afdera for biotechnological applications and adaptation mechanisms, such as RiPP like (Chan and Burrows 2021), NRPS, PKS (Burns et al. 2005), terpene (Medema et al. 2015), NAGGN (Sagot et al. 2010) and ectoine (Jorge et al. 2016). Ectoine, in particular, enables halophiles to endure hypersaline conditions, suggesting that “salt-out” mechanism likely contributes to the survival of microbes in Lake Afdera's high salinity (Ma et al. 2010).

Bacteriocin BGCs were also detected using BAGEL4 (Table S2). This survey has yielded several catalogues of bacteriocin clusters (Fig. 6) with antibiotic potential (Cotter et al. 2013). An earlier survey of hypersaline environments revealed multiple BGCs including bacteriocins, and these clusters were hypothesized to play crucial functions in the survival of microbial community (Crawford et al. 2016, Ziko et al. 2019). It is possible that microbes inhabiting Lake Afdera utilize similar survival mechanisms. Further analysis of the metagenome for C and KS domains, using NaPDoS pipelines, identified antimicrobial peptides such as lichenysin, bacitracin, bacillibactin and fengycin. Lichenysin, renowned for multi-functionality, serve as an efficient ion chelator and surfactant that maintains stability even under extreme conditions (Yeak et al. 2022). Additionally, C domains originating from Gammaproteobacteria aligned with syringomycin, recognized for its antimicrobial and biosurfactant functions (Raaijmakers et al. 2010). Simultaneously, the majority of KS domain sequences were found to closely align with compounds related to fatty acid synthesis (FAS) (Fig. 8). FAS compounds support the survival of organisms in extreme environments by providing photoprotective activity against UV radiation and shielding from ROS (Chen et al. 2020). Consequently, widespread distribution of BGCs plays vital roles in microbial adaptations to high UV radiation, extreme temperatures, salinity and desiccation stress (Wong et al. 2018, Wang et al. 2019).

In this study, metagenome analysis predicted multiple genes conveying metal tolerance in microorganisms. Metals serve a key role within these organisms, acting as catalysts, co-factors for enzymes, stabilizers for proteins and participate in several redox reactions either by donating or accepting electrons (Dopson and Holmes 2014). However, Lake Afdera is typically enriched with high metal concentrations, which can be toxic to microorganisms. Several open reading frames (ORFs) have been identified to code for putative proteins associated with copper resistance. Notably, the multi-copper oxidase, copA, has been detected and found to play an important role in copper detoxification by reducing oxygen to water (Quintanar et al. 2007). Further metagenome analyses unveiled the presence of copper-resistance proteins, CopB, CopC, CopD and CopG, which are recognized for their role in the uptake and transport of copper to cytoplasm for subsequent expulsion (Benison 2019).

Other genes relating to heavy metal tolerance detected in this environment include mercuric reductase (*merA*) and its regulatory proteins. These proteins catalyzes the reduction of Hg(II) to volatile Hg(0), effectively detoxifying the immediate microbial environment (Barkay et al. 2010). The Lake Afdera metagenome also contained *znuABC* genes, which have been identified as involved in the uptake and intracellular regulation of Zn^2+^ ions across the cell membrane (Mikhaylina et al. 2018), and genes encoding for multi-metal resistance protein such as cobalt-zinc-cadmium CzcD and magnesium-cobalt efflux protein CorC. These resistance systems are employed by cells to expel multi-heavy metals out of the cell membrane (Dopson and Holmes 2014).

Metagenome analysis has identified various copies of genes responsible for generation of several anti-oxidative enzymes, which are involved in protecting against oxidative stress. This includes superoxide dismutase, glutathione peroxidase (GPx), glutathione (GSH), catalases, reductases, glutaredoxin and peroxiredoxin (Supplementary Table S6). These enzymes enable bacteria's DNA, proteins, lipid membranes as well as metabolic system and other cell compartments to function efficiently (Kumar et al. 2019, Abdel-Mageed et al. 2020). Among other genes detected for oxidative stress management are *Rbr* and the alkyl hydroperoxide reductase subunit C, which convert endogenously generated hydrogen peroxide into water (Imlay 2013). Other compounds predicted to be effective for the management of oxidative stress include trehalose, sugar molecule known for its role as stress protectant against the damage caused by environmental stresses, such as osmotic stress (Chen et al. 2017).

In hypersaline environments, osmotic stress significantly disturbs the internal osmotic balance of microbial life. Genome mining has identified various genes and solutes that are crucial for neutralizing osmotic stress. Among these key genes *betT, opuAC* and *proW* are responsible for the uptake of choline, glycine/betaine and glycine betaine/L-proline respectively. These genes help to counteract osmotic pressure across the membrane (Kempf and Bremer 1998). In addition, other predicted compounds include peptide such as NAGGN and ectoines (Sagot et al. 2010). The presence of these genes and compounds underscores their potential importance as osmoregulators to counteract osmotic stress at Lake Afdera.

## Conclusion

Our findings present the first metagenomic study that identified BGCs involved in synthesizing various classes of compounds in the microbiome of Ethiopia's Lake Afdera. These include NRPS, NRP-metallophore, bacteriocins, RiPP like and ectoine. These identified BGCs possess a wide range of potential uses in industry and could also hold significant potential in environmental and medicinal fields. Additionally, the study isolated and identified strains of *Bacillus*. The sequencing analysis also highlighted BGCs associated with microbial adaptation to harsh environmental conditions. These genes equip microorganisms to withstand challenges such as salinity, extreme temperatures, high metal concentrations, intense radiation and desiccation. Consequently, the analysis of these BGCs has revealed natural products that could be significant for the environmental adaptation of Lake Afdera microbiome, Afar Depression. Further research is needed to gain a detailed understanding of the molecular mechanisms underlying poly-extremophiles in Lake Afdera and to elucidate the anti-stress mechanisms these organisms employ.

## Supplementary Material

xtae008_Supplemental_File

## Data Availability

The raw metagenomic reads were deposited to Sequence Read Archive (SRA), NCBI and Bio-Sample and SRA accession numbers were received. The accession number PRJNA895852 corresponds to Lake Afdera. The bacterial isolates were archived in the GenBank database (https://www.ncbi.nlm.nih.gov/genbank/) under accession numbers OR230583 and OR230582.
